# The Impact of Coursework Demand and Learning Engagement on Mental Fatigue in Online College Students

**DOI:** 10.3390/ijerph22121860

**Published:** 2025-12-13

**Authors:** Fethi Ahmet Inan, Edwin Teye Sosi, Deniz Unal, Fatemeh Marzban, Gail Alleyne Bayne

**Affiliations:** Educational Instructional Technology, Texas Tech University, Lubbock, TX 79409, USA; esosi@ttu.edu (E.T.S.); dnal@ttu.edu (D.U.); fatemeh.marzban@ttu.edu (F.M.);

**Keywords:** mental fatigue, effort, engagement, workload, task value, well-being, motivation

## Abstract

The purpose of this study was to examine the relationships among coursework demand, course value, learning engagement, and mental fatigue among online undergraduate students in the United States. Data was collected through a panel survey involving 415 online student complete responses across the study variables. Structural Equation Modeling (SEM) was employed to analyze the relationships between the variables of interest. The findings revealed that coursework demand exerted a direct positive effect on mental fatigue, meaning higher demand leads to more fatigue. In contrast, course value indirectly reduced mental fatigue by positively enhancing learning engagement. Furthermore, learning engagement had a direct negative impact on mental fatigue, suggesting that higher levels of engagement may function as a potential buffer against the negative effects of increased coursework demand. While the variables in the current model have been examined individually in prior research, this study explored the mechanisms of the relevant variables and the pathways through which they affect mental fatigue, particularly among online learning populations who are more susceptible to experiencing online learning fatigue. The current findings therefore have significant implications for public health and higher education, suggesting that interventions focused on carefully adjusting coursework demand, increasing course value, and boosting learning engagement could be an effective strategy to mitigate mental fatigue and promote the overall well-being of college students.

## 1. Introduction

Online education is experiencing rapid expansion, offering flexible learning pathways that cater for diverse student needs through a wide array of courses, programs, and innovative pedagogical approaches [1]. The flexibility of online learning, particularly asynchronous courses, is especially appealing to those juggling multiple responsibilities [2]. Despite the numerous benefits and conveniences attributed to online learning, it is not without its challenges, most notably the increased risk of mental fatigue. This heightened fatigue stems from the constant demands of self-motivation, time management, and the lack of traditional classroom interactions [3,4,5,6].

Mental fatigue is characterized by a decline in mental processing performance due to prolonged cognitive engagement [7,8]. It has been established that it has negative effects on attention, memory, decision-making, motivation, task performance, and productivity of individuals [9,10,11,12,13]. While mental fatigue typically does not last for a longer time span, it can significantly impact student academic progress and escalate to severe mental health disorders [11].

Despite these concerns and a growing body of research on mental fatigue in corporate settings [14,15,16,17], its impact on student well-being, particularly in online learning environments, remains under-researched [18]. There is however emerging research evidence [19,20] that revealed a higher prevalence of mental fatigue among online students compared to their in-person counterparts. This alarming trend, coupled with the surge in demand for online learning, underscores the urgent need for further research to understand the sources of mental fatigue. Particularly, studies that examine the course and personal-level factors contributing to this phenomenon, alongside evidence-based strategies to mitigate it, will significantly enrich the academic discourse surrounding the issue. Furthermore, existing studies mainly focus on individual elements in isolation, limiting our understanding of how various factors such as coursework demand, course value, and learning engagement interact and the pathways through which they lead to fatigue among online students.

This study therefore seeks to contribute to the literature by examining how coursework demand, course value, and learning engagement influence mental fatigue among online students, with a particular focus on the direct and indirect effects of coursework demand and course value, and most critically, the mediating role of learning engagement. By shedding light on the direct and indirect sources of mental fatigue, this study aims to inform practitioners on the design of course platforms, content delivery methods, and student workload management strategies, ultimately enhancing the online learning experience of students.

## 2. Literature Review

### 2.1. Theoretical Review

The conceptualization of our research model and hypotheses was grounded on the tenets of the cognitive control theory of fatigue (CCTF). The CCTF, which was propounded by Robert Hockey, seeks to offer basis for the origins of mental fatigue and the role of motivational variables in the control of fatigue [21]. Contrary to earlier human performance improvement theories that attributes fatigue solely to the depletion of energy from demanding mental tasks [8,21], the tenets of the CCTF holds that, an interplay of energy resources and the adaptive human agency for motivational control shapes the occurrence of mental fatigue [8]. In specific terms, the theory holds that engagement, motivation, and self-regulation jointly interrelate to influence mental fatigue outcomes in humans.

In the conceptualization of the theory, engagement is considered as a consequence of task demands [21]. The anticipated marginal benefit of executing a task therefore informs the level of effort and time to be invested. It is argued that as rational beings, humans engage in appraisal of the marginal costs (mental effort and time) versus the marginal benefits of their decisions [7,22,23]. Fatigue causes humans to be aware of the opportunity cost of their current task engagements and the potential utilities from alternative goals [8]. Continuous demonstration of a behavior will therefore proceed when the marginal-benefit evaluation motivates favorable spending of additional energy. More so, in situations where conscious evaluations are not performed, it is argued that an individual’s experience of fatigue may be shaped by a subconscious analysis of cost and benefits to either expend or conserve their energy [22,23]. Considering this, fatigue-induced performance decline may be caused by a reduced desire to exert extra time and mental effort under task-demanding conditions that offer lower levels of utilities relative to anticipated benefits from alternative actions [21,24,25]. To this end, reduced motivation for engagement due to appraised task value is considered as one of the predictors of mental fatigue in humans [26]. In other words, people will not be motivated to engage in task performance when energetical costs are projected to outweigh anticipated rewards from tasks.

Due to the cost–reward analysis among humans, task goals are deemed important in time and effort investment. To this end, even under high task-demanding conditions (e.g., stress, fatigue), compensatory engagement is triggered, leading to an allocation of extra time and cognitive resources [21,26]. The theory further predicts that when competing goals are present, fatigue effects from a task engagement is likely to be greater and vice versa [21]. Hence, Hockey [21] argues that fatigue is a problem of management of effort and time allocation rather than the sole diminishing of energy resources, thereby rendering self-regulation as a quintessential factor in managing fatigue. Fatigue is therefore conceptualized as a negative affective state that one experiences when much time and effort is exerted on a given task that the individual would prefer to disengage and shift existing cognitive resources to an alternative task engagement [8].

Quite recently, Albert Kok offered a neuroscience perspective that supports the thesis that a cost–benefit appraisal in the brain determines cognitive resource allocation, which, in turn, dictates fatigue and subsequent task motivation [27]. Resources are interpreted to encompass the functional state of networks in the brain that interact with cognitive and motivation systems to influence task performance. Fatigue is therefore attributed to these large-scale networks (fatigue networks) that are activated in the brain during effortful tasks performance [27,28,29]. It is explained that when high cost is imposed on the brain’s cortical pathways, it translates into a global reduction in the integration capacity of the brain’s network architecture. In summary, the CCTF suggests that mental fatigue is a consequence of engagement–reward imbalance. That is, when the time and mental effort required are proportionally greater than the associated learning outcome (reward), motivation for the task decreases, and mental fatigue sets in [30]. During times of mental fatigue, shifts in motivation tend to drive performance rather than mere depreciation of a finite mental energy. Based on the CCTF, the present study examines the impact of course value, coursework demand, and learning engagement on fatigue experienced in online learning environments.

### 2.2. Learning Engagement

Learning engagement, which encompasses both mental effort and time commitment to learning, has garnered considerable attention in online learning environments [31,32,33]. Engagement is associated with positive outcomes, such as increased motivation, satisfaction, and academic performance [34,35,36]. However, it also demands significant mental effort and time commitment. Research, primarily conducted in lab settings with short instruction durations, suggests that heightened mental effort and extended study periods can result in reduced performance and increased mental fatigue [37,38].

Given that learning engagement often requires substantial mental effort and time, it is reasonable to hypothesize that higher engagement may lead to increased mental fatigue. However, this claim largely applies to settings, where tasks are specifically designed to induce fatigue in a short amount of time. Additionally, such studies often do not account for the individual’s background, interests, or motivations when exploring the link between increased engagement and mental fatigue. While higher task demands typically correspond to higher fatigue levels, external and internal motivation elements has been shown to reduce fatigue across subjective, behavioral, and psychophysiological measures [10,21,26,39]. Milyavskaya and her colleagues [40] also found that despite expending more effort, students experienced less fatigue when the task aligned with their interests. In contrast to lab-based research context, where tasks are designed to induce fatigue within a constrained time frame, online learning environments offer more flexibility, though students are still bound by deadlines. Therefore, we propose that, in the context of online courses, higher learning engagement may not lead to increased mental fatigue.

**H1.**  *Higher learning engagement decreases students’ experience of mental fatigue.*

### 2.3. Course Value

Previous research highlights the role of course value, conceptualized as an individual’s personal interest in the course topics and their perception of its relevance to success and growth, in promoting learning engagement [41,42]. Studies have consistently shown that subjective value of the task directly influences learners’ decisions, effort, persistence, and overall engagement [43,44]. Empirical evidence also supports the connection between course value and learning engagement. Johnson and Sinatra [45], and Cole et al. [46] found that value of the academic task was associated with higher learning engagement and effort. In particular, course value, which includes utility and performance values, was predictive of learning engagement. Edwards and Dai [47] further solidified this connection by showing the significant influence of course value on both behavioral and learning engagement of college students. Recent studies on online learning have confirmed these findings. Sun et al. [48] found that students’ perceived value of course goals positively influence their engagement in online learning. Similarly, it has been found that students’ interest in the subject area leads students to engage in the task and invest more time and effort [40]. Building upon this robust empirical evidence, the following hypotheses were proposed:

**H2.**  *Course value positively impacts learning engagement.*

**H3.**  *Course value indirectly impacts mental fatigue through learning engagement.*

### 2.4. Coursework Demand

Coursework demand is conceptualized as the workload, difficulty, and pace of activities, content, and tasks that students are expected to complete for a given class. Higher coursework demand has been consistently linked to negative outcomes, including mental fatigue [49,50,51]. This is particularly relevant in online learning environments, where students face unique challenges such as self-regulation and limited external support. For example, Sy and colleagues [50] indicated that demanding coursework significantly contributes to the experience of mental fatigue among online college students. High demand directly imposes greater cognitive load on students [52,53]. This sustained cognitive load can deplete mental resources, leading to mental fatigue [19,54]. While individuals may attempt to compensate for increased load by allocating additional time and cognitive resources [55], this response may be insufficient or unsustainable in the face of excessive demands. Moreover, the increased effort and time investment required to manage a high coursework load may inadvertently contribute to mental fatigue. We therefore hypothesize that:

**H4.**  *Higher coursework demand directly and positively impacts mental fatigue.*

**H5.**  *Coursework demand indirectly impacts mental fatigue through increased learning engagement.*

## 3. Methodology

### 3.1. Hypothesized Model

This study’s conceptual model proposes a relationship between coursework demand, course value, learning engagement, and mental fatigue in online learners. This framework, built upon previous research, highlights the complex interplay of factors contributing to mental fatigue in online learning. Within the model, a positive impact is indicated when two variables move in the same direction; when the value of one increases, the other also increases. Conversely, a negative impact is realized when the increase in value of one variable is associated with a decrease in the other, indicating movement in opposite directions. It is important to note that these terms do not imply whether the impact is good or bad; they simply describe the direction of the relationship between variables.

**Figure 1:**
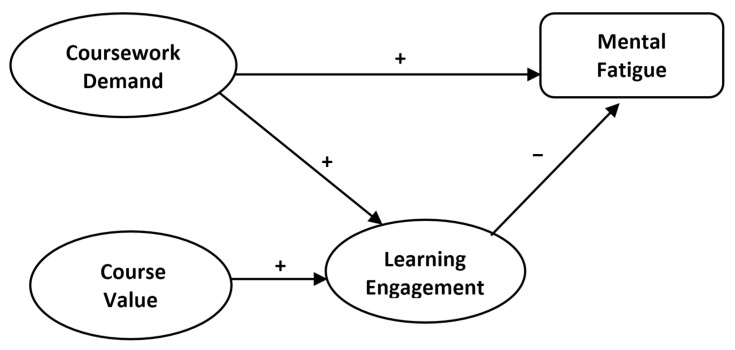
Proposed Conceptual Model. Plus (+) and minus (−) signs indicate positive and negative relationships between variables.

The model hypothesizes that higher levels of learning engagement will directly and negatively impact mental fatigue. This means that when learners invest more effort and time in course materials and activities, they experience less mental fatigue. Additionally, the model suggests that course value will positively influence learning engagement; that learners will put more time and mental effort into course activities if they value the course. This, in turn, will indirectly decrease their experience of mental fatigue. On the other hand, coursework demand is expected to have a direct and positive impact on mental fatigue because the higher workload, faster pace, and greater difficulty of coursework require higher cognitive processing, resulting in increased mental fatigue. The coursework demand also indirectly influences mental fatigue by increasing learning engagement. A key element of this model is the mediating role of learning engagement, which is posited to connect coursework demand and course value with mental fatigue. Figure 1 illustrates the proposed conceptual model of relationships among coursework demand, course value, learning engagement, and mental fatigue.

### 3.2. Research Design

This study employed a quantitative, non-experimental correlational research design, utilizing a cross-sectional panel survey approach [56,57,58]. This design was selected to examine the hypothesized relationships among coursework demand, course value, learning engagement, and mental fatigue. The cross-sectional survey method offered an efficient and cost-effective means of capturing a snapshot of these dynamics within a large, geographically dispersed population of online students [57,59]. While this design allows for the identification of associations among variables, its cross-sectional nature precludes the inference of causal relationships.

### 3.3. Instruments and Measures

Data was collected through online questionnaires administered to students. The questionnaire comprised several sections employing various instruments to assess the hypothesized model. Students’ self-reported mental fatigue during online courses was measured using the Student Mental Fatigue Scale [19]. An average score was calculated from responses to eight items on a 5-point Likert scale (strongly disagree to strongly agree), with the scale demonstrating high reliability (Cronbach’s α = 0.91). Two indicators were used to generate the latent construct of learning engagement. Mental Effort was measured using Paas’ [60] 9-point mental effort rating scale (extremely low to extremely high), which has demonstrated high reliability (Cronbach’s α = 0.9) and has been widely used in prior research [60,61]. Time Commitment was measured through student self-report of hours per week required on course activities outside of class [60]. Coursework demand was generated using three indicators: course difficulty, course workload, and course pace. The items were adapted from the Student Evaluations of Educational Quality [62] which were ranked on Likert scales (e.g., very easy-very hard for difficulty, very light-very heavy for workload, too slow-too fast for pace). Overall, this scale demonstrates strong reliability, with an average Cronbach’s alpha of 0.88 [62]. Course Value was generated using two indicators. Subject Interest was measured with a single item on a 5-point Likert scale (very low to very high) asking students to rate their interest in the course subject. Relevance was measured using a six-item relevance subscale [19] with high internal reliability (α = 0.81).

### 3.4. Participants

The study sample consisted of undergraduate students enrolled in online courses at colleges and universities across the United States. Inclusion criteria required that participants be currently enrolled as undergraduate students (either part-time or full-time), be at least 18 years of age, and be enrolled in at least one online course. Recruitment was conducted through Qualtrics Panel LLC, which distributed the survey to potential respondents from its panel pool and provided incentives to encourage participation. To. The panel was proportionally structured to reflect the general characteristics of the U.S. undergraduate student population. Participants represented a wide range of academic disciplines and institutions. ensure data integrity, participation frequency was monitored, and each respondent was permitted to complete the survey only once.

Participants were given an online survey and asked to self-report their perceptions of their level of mental fatigue and their views on the course environment while taking an online course. 415 students with complete data on the study variables were included in the analysis. The students’ ages ranged from 18 to 50 years (M = 27.0, SD = 7.5). The sample consisted of a larger proportion of women (82.9%) than men (17.1%). The ethnic composition consisted of the following ethnicities: White (65.8%), Black/African American (15.4%), Hispanic/Latino (10.6%), Asian (3.4%), Multiple (2.2%) %), American Indian/Alaskan Native (1.7%), Other (1.0%). About two-thirds of participants were employed as part-time (30.6%) or full-time (33.0%) workers.

### 3.5. Data Analysis

The latent variable structural equation modeling (SEM) was used to estimate the hypothesized relationships among our multiple constructs and students’ experience of mental fatigue [63,64]. Unlike traditional multivariate analysis techniques that assume that variables are measured without error [65], the SEM approach considers potential measurement errors [66]. By applying the latent variable SEM approach, we were also able to test for the measurement model along with estimating our structural model [67], thereby granting us confidence of attainment of precise and robust estimates [68]. Mplus statistical software (Version 8.11) was utilized to estimate the Structural Equation Model. The analysis employed the Maximum Likelihood (ML) estimator, which was chosen for its ability to provide robust parameter estimates and its suitability for continuous observed variables, consistent with the scales used in this study [63].

## 4. Results

### 4.1. Model Assessment

In this study, we utilized a structural equation model (SEM) to explore the relationships between course value, coursework demand, engagement, and mental fatigue. The proposed model consisted of both latent and observed variables, allowing for a comprehensive investigation of the hypothesized relationships. We employed the maximum likelihood parameter estimation method to evaluate the proposed model and determine the statistical significance of the hypothesized direction and magnitude of relationships between variables. For each predictor, we estimated the magnitude, direction, and significance of both direct and indirect effects. This combined approach provided a unified framework for estimating both the measurement model (describing how latent variables are measured by indicators) and the structural relationships among latent variables. This approach is preferred as it offers a comprehensive understanding of how latent constructs are measured and interconnected with other variables within the same model [69].

The model fit was evaluated using multiple indices established to guide SEM estimations [63,70]. Conventionally, with reference to the Comparative Fit Index (CFI) and Tucker–Lewis Index (TLI), values that are greater than 0.90 are considered acceptable, and values above 0.95 indicate good model fit [61]. Root Mean Square Error of Approximation (RMSEA) and Standardized Root Mean Square Residual (SRMR) values below 0.08 reflect acceptable fit whereas relatively lower values below 0.05 reflect excellent model fit [70]. In the present study, the fit indices obtained fall within these established thresholds, supporting the overall adequacy of the proposed structural model fitness to the data. The fit indices were excellent, with a CFI of 0.976 and a TLI of 0.959. Furthermore, the other fit indices confirmed a strong model, with an RMSEA of 0.045 and an SRMR of 0.045.

### 4.2. Model Estimates

The model incorporated several latent constructs. Coursework demand was measured as a latent variable composed of three indicators: course pace, difficulty, and workload. These factors significantly loaded onto the latent variable (loadings of 0.516,0.666, and 0.740, with *p* < 0.001), confirming that they adequately represent students’ perceptions of workload. Course value was also modeled as a latent variable, represented by the perceived relevance of the subject matter and students’ personal interest in the course. Both indicators loaded onto the latent construct (loadings of 0.699 and 0.727, with *p* < 0.001), indicating that course value reflects both students’ interest in the subject matter and the perceived relevance of the course to their academic or career goals. Learning engagement was captured through students’ mental effort and the time committed to the course. The indicators for Learning Engagement demonstrated acceptable standardized factor loadings (range: 0.447 to 0.448, with *p* < 0.001), confirming that this construct reflects the level of time allocation and cognitive investment in the course. While these factor loadings appear to be moderate, their magnitudes exceed the conventional thresholds for factor inclusion, which typically begin at 0.30 [66,71]. Moreover, as stipulated by Hair and colleagues [72], the utilization of a larger sample size offers the statistical power required to robustly accept factor loadings of 0.3 and above. Mental fatigue was directly measured through a validated self-report instrument and included as an observed variable in the model.

**Figure 2:**
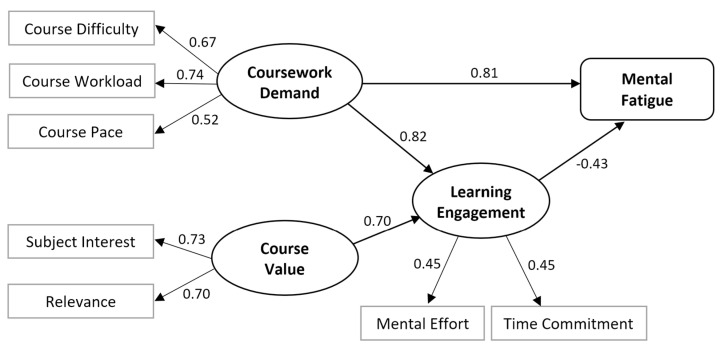
Estimated Model.

The structural model estimates revealed significant relationships among latent and observed variables (see Figure 2). Overall, the hypothesized factors explained approximately 32% of the variance in student mental fatigue. Consistent with the conceptual model, coursework demand had a direct positive effect on mental fatigue (standardized β = 0.807, *p* < 0.001), indicating that increased demand contributed to higher levels of mental fatigue. However, coursework demand also positively influenced learning engagement (standardized β = 0.820, *p* < 0.001), and its indirect negative impact through this mediating pathway (standardized β = −0.349, *p* < 0.001) partially offset its direct positive effect on mental fatigue. This suggests a complex interplay where coursework demand stimulates learning engagement, and the resulting engagement may help buffer its direct, fatigue-inducing impact. Course value, on the other hand, positively influenced learning engagement (standardized β = 0.701, *p* < 0.001), indicating that students who perceive their courses as valuable are more likely to engage deeply with the course material and activities. The course value indirectly reduced mental fatigue through learning engagement (standardized β = −0.298, *p* < 0.001), emphasizing the motivational importance of course value in fostering engagement and mitigating fatigue. Learning engagement itself was a key factor in the model, showing a significant negative direct effect on mental fatigue (standardized β = −0.425, *p* < 0.001), suggesting that higher learning engagement reduces the experience of mental fatigue.

The finding sheds light on the complex interplay between workload, value, and engagement in online courses. Table 1 contains the standardized regression coefficient (beta) for endogenous variables, which represents the direction and magnitude of the estimated effect between the variables.

## 5. Discussion

### 5.1. Learning Engagement and Mental Fatigue

Our results revealed that students’ learning engagement had a statistically significant negative relationship with mental fatigue. This suggests that students who were more actively involved in their studies, dedicating higher mental effort and time to course tasks, tend to report lower levels of mental fatigue in online courses. While this finding may appear to contradict well-known principles of cognitive load theory [73] and some of the previous research findings [37,38,74], it is not entirely unexpected when considering the limitations of these previous studies. The majority of current research investigating mental fatigue has utilized controlled laboratory settings with time-restricted tasks intentionally structured to induce fatigue [75,76,77]. In contrast, the self-paced feature of online learning environments may offer mental rejuvenation time-break for students involved in higher task-engagement [78]. Our results are consistent with recent studies such as the work of Milyavskaya and her colleagues [40], who found that increased effort does not necessarily lead to increased mental fatigue when learners have control over choice of task type and time commitment. Further, the work of Cotton et al. [79], who examined the occurrence of mind-wandering, mental fatigue and task disengagement in both online and face-to-face learners, provided strong evidence that studying remotely does not necessarily lead to increased cognitive load, especially when learners’ task engagement is high. Further, our finding can be explained by the cognitive control fatigue theory, which posits that the anticipated fatigue from higher task demands can be offset by the perceived benefits of increased engagement [7,22,55]. This supports the view that fatigue is not solely a result of depleted cognitive resources but is influenced by the interplay of motivation and cognitive control [80,81,82,83]. Given that, it is plausible that higher motivation to learn contributed to reduced mental fatigue, even when students invested more effort and time in their studies [21,84].

### 5.2. Course Value, Learning Engagement, and Mental Fatigue

Our second hypothesized path explored the relationship between course value and student learning engagement. Statistical evidence suggests a significant positive relationship, indicating that students who find a course relevant and interesting are likely to be more engaged. This aligns with expectancy-value research, which suggests that students exert more time and effort when they find the content interesting and relevant to their personal or professional goals [43,44]. Earlier studies by Johnson and Sinatra [45] also support this association, highlighting the link between task value and cognitive engagement and effort investment. While empirical evidence in online learning is limited, Sun et al. [48] found a similar positive impact of task value on online learning engagement. The inclusion of subjective interest in the course value construct further strengthens this connection, as motivation research consistently demonstrates the importance of personal interest in promoting effort and engagement [85].

Moreover, the indirect impact of course value on mental fatigue through engagement reinforce recent findings by Milyavskaya et al. [40], who observed that students who are interested in a subject are more likely to choose challenging tasks, dedicate more time and effort, and experience less fatigue despite their increased exertion of mental effort. Overall, our findings underscore the importance of motivation in managing mental fatigue. Students with higher motivation to learn, driven by course relevance and personal interest, may experience less fatigue even when investing significant effort and time [86]. This suggests that effective task selection and intrinsic motivation may play a crucial role in alleviating mental fatigue, challenging the traditional notion that effort is inherently aversive [82].

### 5.3. Coursework Demand and Mental Fatigue

Students in our study with heavier coursework demand reported higher levels of mental fatigue. Our findings align with previous research indicating that heavier coursework significantly increase mental fatigue [87]. When students face demanding coursework, they may struggle to find time for assignments, experience increased anxiety, and feel overwhelmed by the combined pressure of academic and other commitments. The demanding coursework creates a heightened mental load, requiring more significant effort and potentially leading to resource depletion and fatigue [14]. These factors, coupled with the rapid depletion of mental resources during cognitively demanding tasks, contribute to the increased mental fatigue.

Our results suggest that higher coursework demand is statistically associated with greater mental fatigue, but this effect is mitigated by higher levels of learning engagement, which plays a mediating role in this relationship. Specifically, the results suggest that when students are more actively involved in their learning, even with demanding coursework, they may experience less fatigue [83]. This finding can be attributed to the tenets of the Cognitive Control Theory of Fatigue (CCTF), which posits that humans possess an innate compensatory control mechanism that can motivate them to allocate more resources when they anticipate a higher benefit from completing a given task [21,88]. This compensatory allocation leads to higher levels of task engagement. Crucially, this engagement can be protective against fatigue because the student is motivated to expend effort and time due to the perceived value of the task [40].

## 6. Implications for Practice

This study’s findings offer valuable insights for educators, instructional designers, and administrators aiming to mitigate mental fatigue and enhance the online learning experience of learners. The level of mental fatigue experienced by students is influenced by various factors, including coursework demand, course value, and learning engagement, in addition to individual factors such as external commitments, prior experience, and sleep patterns [14,19,40,83,89,90]. By implementing effective strategies [89], educators can create online learning environments that are both engaging and supportive, promoting student well-being and academic success.

The study suggests a critical role of learning engagement in mitigating mental fatigue. Fostering engagement emerges as one of the key strategies that may be instrumental in reducing mental fatigue. While our cross-sectional design cannot establish causality, the significant negative association suggests that fostering learning engagement is a potential buffer against the negative effects of heavy coursework on mental fatigue. To increase student effort and time commitment, educators should implement learning strategies that promote active participation. This can be achieved by encouraging students to take active roles through project-based learning or assignments that require applying information within authentic contexts [91]. Furthermore, effective instructor facilitation, personalized learning experiences, and individualized support, including progress monitoring and timely assistance, can foster a positive and supportive online learning community, thereby enhancing student engagement [92,93].

The findings also emphasize the motivational power of relevance and interest in online learning. Courses that emphasize relevance and autonomy are associated with higher levels of cognitive engagement, deeper learning, and reduced mental fatigue [40,82,91]. To enhance perceived course value and foster meaningful engagement, design strategies should prioritize aligning course content and assignment tasks with students’ personal and professional objectives. This can be achieved by incorporating real-world applications, offering choices in assignments, and tailoring activities to connect with students’ interests and future career aspirations [94,95]. Specifically, practical implementation may involve curriculum planning that aligns with industry needs, the use of adaptive technologies to individualize learning tasks, and reflective assignments that encourage students to connect course concepts to their future professional goals [89,96,97,98].

While heavier coursework can indirectly increase learning engagement, its direct impact on mental fatigue is substantial, necessitating careful management of coursework demand. Therefore, courses should be designed with manageable workload, difficulty, and pace, considering both cognitive demands and students’ time constraints. To address diverse student needs, strategies such as frequent breaks, pacing options, and self-paced learning opportunities should be integrated [89]. Notwithstanding these recommendations, it is worth noting that striking a balance is crucial, as easy courses can lead to decreased perceived value, disinterest and disengagement [99,100]. Additionally, providing appropriate support mechanisms such as personalized feedback, mentoring, and scaffolding can enable students to handle heavier coursework while minimizing the experience of increasing mental fatigue [101]. By designing challenging yet engaging courses and providing adequate student support, educators can create online learning experiences that are both effective and enjoyable.

## 7. Limitations

While this study significantly contributes to the academic literature on mental fatigue in online learning environments, several limitations ought to be acknowledged. First, it is crucial to interpret the findings within the constraints of the quantitative, non-experimental correlational research design. Because data were collected at a single point in time, the study is limited to examining associations and potential influences among the variables rather than establishing definitive causal relationships [56,57]. Furthermore, although the structural model is built on research and theory to test the proposed directionality of relationships, the Structural Equation Model (SEM) analysis is inherently restricted by the correlational nature of the data and thus does not show actual causality or confirm the direction of the effects [64]. Therefore, it is important to interpret the calculated path coefficients as measures of association. Future research utilizing a longitudinal or experimental design is recommended to confirm the directionality of these complex relationships. Future studies would also benefit from employing longitudinal designs to examine how mental fatigue fluctuates throughout the semester and to investigate the long-term effects of mental fatigue on learning outcomes and performance.

Additionally, the study relied on self-reported measures, which may introduce bias due to subjective perception. Individual students may interpret the survey items differently based on their personal context, standards, or external responsibilities, leading to variations in perceived scores that are not strictly related to the construct being measured [102]. Future studies should address this subjectivity by integrating objective behavioral or physiological data. For example, learning engagement can be captured through student activities recorded in online log data, while mental fatigue can be captured by objective physiological measurements, such as eye tracking or EEG, to complement self-reports and provide a more accurate and comprehensive data [103,104].

It is essential to explore how coursework can be divided into manageable units without sacrificing learning outcomes. This would involve finding optimal ways to balance cognitive load and pacing across online course structures. While this study suggests that certain aspects of coursework demand lead to mental fatigue, it is important to explore the effects of different online course designs and technologies on mental fatigue [89]. For example, future studies should investigate factors such as synchronous versus asynchronous formats, project-based versus lecture-based activities, and technologies like video conferencing and virtual reality influence mental fatigue. Additionally, factors related to platform usability design such as navigation, visual layout, and accessibility should be examined to assess their role in contributing to mental fatigue.

With regards to the operationalization of learning engagement, it should be conceptualized as a complex multidimensional construct encompassing cognitive, behavioral, and emotional elements. The current study, however, defined and measured engagement narrowly as a construct reflecting only time commitment and the mental effort invested by online students in their course activities. This operational limitation, which is reflected in the modest factor loadings for the learning engagement construct, suggests that our measurement may have captured only a limited aspect of this broader complex construct. Therefore, we recommend that future studies utilize a more comprehensive measure to explore and integrate other dimensions of learning engagement. Specifically, this includes objective measures derived from student activity logs from the learning management system (e.g., frequency of logins, clicks, or discussion forum participation); dynamic indicators that capture real-time mental processing (e.g., eye-tracking data or trackers to monitor cognitive load); and measures assessing students’ emotional responses during learning (e.g., feelings of boredom, enjoyment, or anxiety toward the course material and activities) [105,106,107,108].

The study sample exhibited a significant demographic imbalance that may limit the generalizability of our findings. The sample included a substantially larger proportion of female participants compared to male participants, which is consistent with the documented trend of higher female enrollment in online higher education [109,110]. Therefore, this gender imbalance and the presence of non-traditional student factors should be considered when interpreting our findings. Within this population, extraneous factors such as health conditions, prior experience, employment, family responsibilities, and self-regulation abilities may significantly influence the perceived experience of fatigue and learning engagement [111,112,113,114]. In addition to more balanced future samples, we recommend that future research examine individual difference variables. This includes exploring the relationship between self-regulation and mental fatigue in online environments, examining the effects of course design elements and effective teacher facilitation on student learning engagement and mental fatigue, and investigating the coping strategies students use to minimize fatigue.

## 8. Conclusions

This study unravels the complex interplay of coursework demand, course value, engagement, and mental fatigue in online learning, offering valuable insights for educators and instructional designers. The findings provide practical guidance for creating mentally friendly learning environments that minimize fatigue and enhance the overall learning experience. Notably, the research underscores the importance of transitioning from merely identifying sources of mental fatigue to proactively implementing preventive and recovery strategies to mitigate its occurrence and impact. The proposed model and its findings serve as a valuable framework for future research, guiding exploration of the interactions between mental fatigue and learning outcomes, as well as relevant factors such as student commitments, coping mechanisms, social support, and resource access. By proactively exploring these factors and interventions, the negative effects of mental fatigue can be counteracted, fostering healthier learning experiences for online students.

## Figures and Tables

**Table 1:** **Direct and Indirect Effects of Factors**.

	Endogenous (Dependent) Variables			
Variables	Learning Engagement	Mental Fatigue		
	Direct	Indirect	Direct	Total
1. Course Workload	0.820 *	−0.349 *	0.807 *	0.458 *
2. Course value	0.701 *	−0.298 *	--	−0.298 *
3. Learner Engagement	--	--	−0.425 *	−0.425 *

Note: All coefficients are standardized and * *p* < 0.001.

## Data Availability

The data that supports the findings of this study can be obtained from the corresponding author upon reasonable request.
